# Supplementary material for: Oral Magnesium Supplementation for Treating Glucose Metabolism Parameters in People with or at Risk of Diabetes: A Systematic Review and Meta-Analysis of Double-Blind Randomized Controlled Trials
Source: Nutrients. 2021 Nov 15;13(11):4074. doi: 10.3390/nu13114074 (PMC8619199; doi:10.3390/nu13114074)
Supplement: Supplementary file 1 [file nutrients-13-04074-s001.zip › nutrients-1423787-supplementary.pdf]

Table S1. Descriptive characteristics of the studies including diabetic people.

Author (Year)	Country	Glucose Metabolism Parameters	Condition	Study Design	Diabetic Treatment	Daily Mg Dosage	Follow-up (Weeks)	Jadad's Scale	Treated	Age (SD)		Females	BMI (SD)		Placebo	Age (SD)		Females	BMI (SD)	
Corica et al., 1994	Italy	FPG, HbA1c	Type 2 diabetes	Parallel	Diet and sulphonylureas	Mg pidolate 4.5 g	4	3	26	63	5	15			17	61	3	10		
de Lourdes Lima et al., 1998	Brazil	FPG, HbA1c	Type 2 diabetes	Parallel	Oral agents	Mg oxide 41,4 mmol	4	4	39	51.2	11	31	25.5	6.5	54	55.5	8.3	41	25.5	6.5
de Valk et al., 1998	The Netherlands	FPG, HbA1c	Type 2 diabetes	Parallel	Use of insulin	15 mmol Mg-aspartate-HCl	12	4	25	63	8.2	9	28.7	2.7	25	62	7.3	13	27.1	2.7
Eibl et al., 1995	Norway	HbA1c	Type 2 diabetes	Parallel	Oral agents	Mg citrate 30 mmol	12	4	18	63	8	7	27.5	3.2	20	54	1.5	12	29.3	5
Gullestad et al., 1994	Norway	FPG, HbA1c	Type 2 diabetes	Parallel	Mixed	Mg lactate-citrate 15 nmol	16	4	29				25.4	3.7	25				25.3	4.1
Hagg et al., 1999	Sweden	HbA1c	Type 1 diabetes	Parallel	Insulin	Mg hydroxide 250 mg	48	4	15	40.5	8.9	7	24.3	2.5	13	36.9	10.6	8	23.9	3.2
Jamilian et al., 2017	Iran	FPG	Gestational diabetes	Parallel	Only diet	Mg oxide 250 mg	6	5	20	27.8	3.4	20	26.1	1.9	20	27.1	4.9	20	27.4	3.2
Navarrete-Cortes et al., 2014	Mexico	FPG, insulin, HbA1c, HOMA-IR	Type 2 diabetes	Crossover	Oral agents	Mg lactate 750 mg daily (=360 mg elementary Mg)	12	5	56	52.8	8.4	36	30.6	5.7	56	52.8	8.4	36	30.5	5.8
Paolisso et al., 1994	Italy	FPG, insulin	Type 2 diabetes	Crossover	Oral agents	Mag 2 3 g	4	4	8	67.3	4.8	4	30.5	2.5	8	67.3	4.8	4	30.5	2.5
Purvis et al., 1994	USA	FPG	Type 2 diabetes	Crossover	Oral agents	Mg chloride 384 g/day	6	5	28						28					
Rodriguez-Moran et al., 2014	Mexico	FPG, HbA1c, insulin, HOMA-IR	Type 2 diabetes	Parallel	N/A	Mg chloride 2.5 g	16	5	32	59.7	8.3		27.6	9.1	31	54.1	9.6		28.6	4.2
Sadeghian et al., 2019	Iran	FPG, HbA1c, insulin, HOMA-IR	Type 2 diabetes	Parallel	Oral agents and/or insulin	Mg oxide 250 mg	12	5	40	41.2	8.8	27	31.2	5.5	40	42.8	8.4	26	30.9	4.4
Solati et al., 2014	Iran	FPG, 2hOGTT, HbA1c, HOMA-IR, insulin,	Type 2 diabetes	Parallel	Metformin, glibenclamide	MgSO4 (300 mg equivalent)	12	5	25	46.7	9	18	26.2	2.9	22	50.2	6.93	16	26.9	5.2

**Total**	**11: type 2; 1: type 1; 1: pregnancy**	**10: parallel; 3: crossover**	**Median = 12 (range: 4–48)**	**Media n = 4**	**361**	**50.6 ± 7.6**	**53.0%**	**27.5 ± 4.4**	**359**	**49.9 ± 6.5**	**57.2 %**	**27.6 ± 4.3**	

Abbreviations: BMI—body mass index; Mg—magnesium; PLC—placebo; SMD—standardized mean difference; FPG—fasting plasma glucose; OGTT—oral glucose tolerance test; HbA1c—glycosilated haemoglobin; HOMA-IR—homeostatic model assessment-insulin resistance; SD—standard deviation.

Table S2. Descriptive characteristics of the studies including people at higher risk of diabetes.

Author (Year)	Country	Glucose Metabolism Endpoint Measures	Condition	Study Design	Mg Daily Dosage	Follow-up (Weeks)	Jadad's Scale	Treated	Age (SD)		Females	BMI (SD)		Placebo	Age (SD)		Females	BMI (SD)	
Alizadeh et al., 2021	Iran	FPG, insulin, HOMA-IR	Policistic ovary syndrome	Parallel	250 mg Mg oxide	8	5	21	26.6	4.9	100	27.8	3.2	20	26.2	5.7	100	26.9	3.8
Chacko et al., 2011	USA	Insulin, C-petide, HbA1c, FPG	Overweight	Crossover	500 mg citrate Mg	4	5	13	47	13.8	4	28.3	4	13	41.9	12.7	6	28.1	5.5
Cunha et al., 2017	Brazil	FPG, insulin, HOMA-IR	Overweight and hypertension	Parallel	Chelate Mg 600 mg	24	4	17	54	7	17	29.7	4.1	18	57	5	18	26.8	3.9
de Souza et al., 2014	Brazil	FPG, insulin, HOMA-IR	Metabolic syndrome	Parallel	Chelate Mg 400 mg	12	3	30	44.6	9.7		33.7	6.7	32	46.6	12.3		34.9	6.1
Farsinejad-Marj, 2021	Iran	FPG, insulin, HOMA-IR, QUICKI	Policistic ovary syndrome	Parallel	250 mg Mg oxide	8	5	30	26.3	3.9	100	27.8	5.1	30	26.0	5.1	100	27.7	5.4
Guerrero-Romero et., 2015	Mexico	Fasting insulin, HOMA-IR, FPG, 2hOGTT	Prediabetes and hypomagnesemia	Parallel	30 mL of MgCl2 = 382 Mg equivalent	16	5	59	42.2	9.5		30.6	6.4	57	42.3	8		31.7	4.7
Joris et al., 2017	The Netherlands	FPG, insulin, HOMA-IR	Overweight	Parallel	Mg citrate 350 mg	24	5	26						26					
Lee et al., 2009	South Korea	FPG, insulin, HOMA-IR	Overweight	Parallel	300 Mg Oxide	12	4	75	36.9	7.9	30	26.7	2.8	80	40.5	7.3	40	26.1	2.3
Mooren et al., 2011	Germany	FPG, 2h OGTT, HOMA-IR, insulin, 2h insulin	Overweight	Parallel	Mg aspartate-hydrochloride 365 mg	24	3	25	30-70			30.9	1.2	22	30–70			30	2.3
Rodrıguez-Moraan et al., 2018	Mexico	FPG	Metabolic syndrome	Parallel	30 mL of MgCl2 = 382 Mg equivalent	16	5	100	39.4	9.8	34	29.7	6.5	98	40.4	10.6	35	30.5	5.9
Simental-Mendiaet al., 2014	Mexico	Fasting insulin, HOMA-IR, FPG, 2hOGTT	Metabolically obese normal weight	Parallel	30 mL of MgCl2 = 382 Mg equivalent	16	5	24	31.9			22.4	1.6	23	39.5			22.7	1.9
Toprak et al., 2017	Turkey	FPG, HbA1c, HOMA-IR	Prediabetes, chronic renal failure and hypomagnesemia	Parallel	365 mg Mg Oxide	12	5	57	55.6	13.8		33.9	2.8	61	57.1	12.9		34.0	2.6

**Total**		**5: overweight; 2:metabolic syndrome; 2: prediabetes; 2: polycystic metabolic syndrome; 1: metabolically obese normal weight**	**11: parallel; 1: crossover**		**Median = 14 (range: 4–24)**	**Median = 5**	**477**	**42.5 ± 10.2**	**42 %**	**28.8 ± 3.8**	**480**	**45.6 ± 10.0**	**50 %**	**28.9 ± 3.8**

Abbreviations: BMI—body mass index; Mg—magnesium; PLC—placebo; SMD—standardized mean difference; FPG—fasting plasma glucose; OGTT—oral glucose tolerance test; HbA1c—glycosilated haemoglobin; HOMA-IR—homeostatic model assessment-insulin resistance; SD—standard deviation.

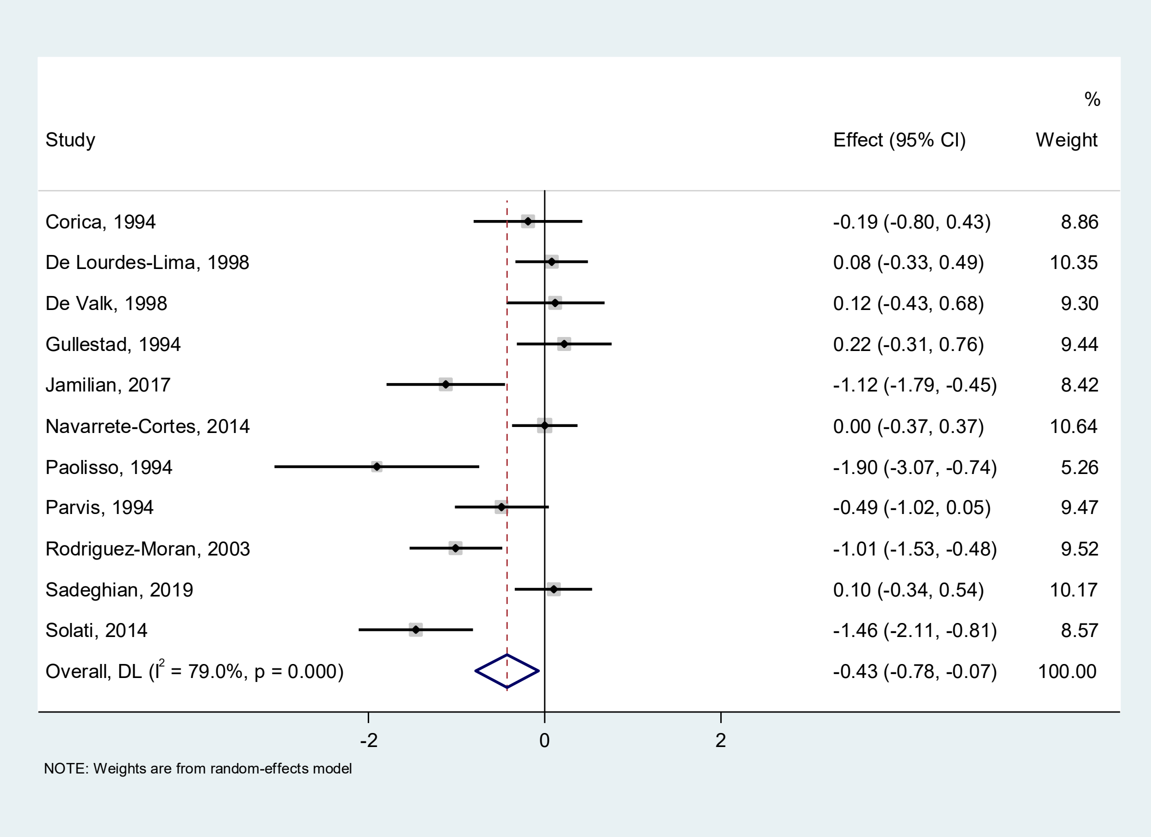

Figure S1. Forrest plots of significant results with oral Mg supplementation vs. placebo in improving fasting plasma glucose in diabetes.

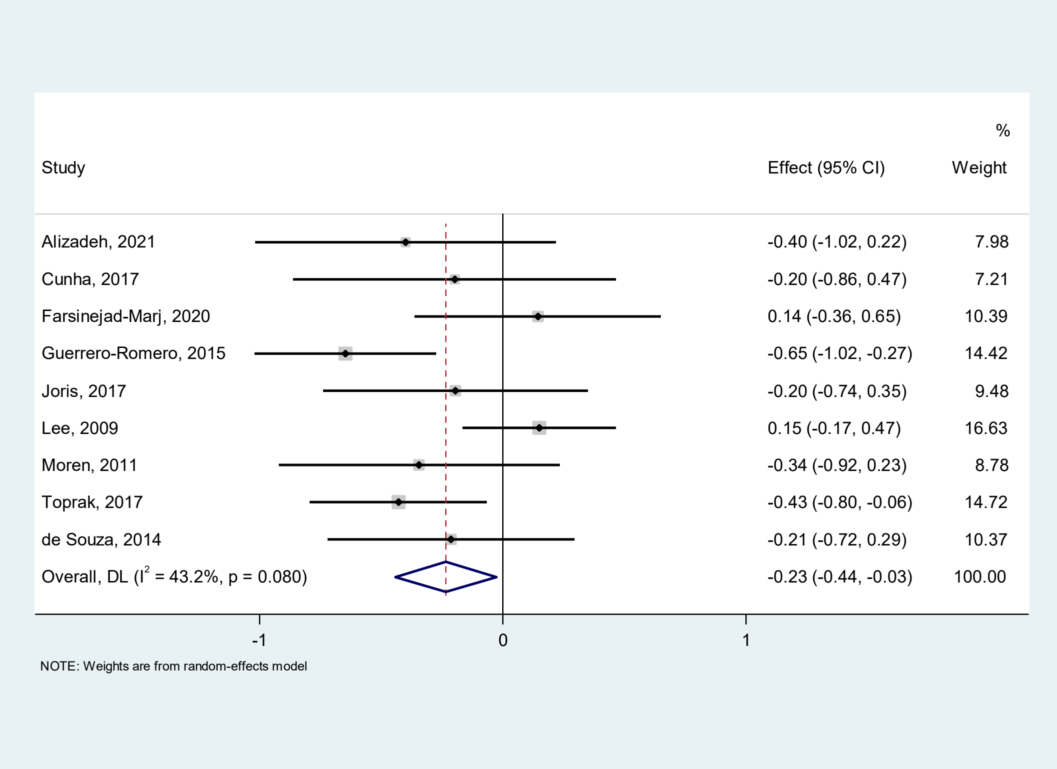

Figure S2. Forrest plots of significant results with oral Mg supplementation vs. placebo in improving fasting plasma glucose in people at high risk of diabetes.

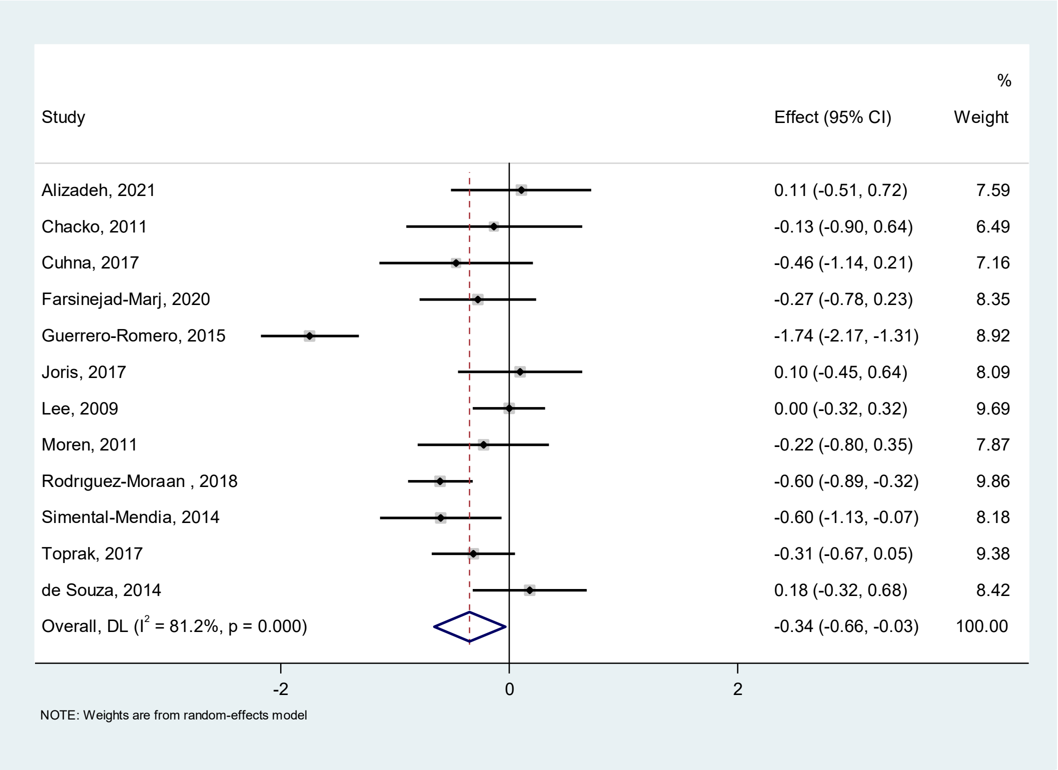

Figure S3. Forrest plots of significant results with oral Mg supplementation vs. placebo in improving HOMA-IR in people at high risk of diabetes.
